# Apoptosis-associated biomarkers in tuberculosis: promising for diagnosis and prognosis prediction

**DOI:** 10.1186/1471-2334-13-45

**Published:** 2013-01-28

**Authors:** Chin-Chung Shu, Ming-Fang Wu, Chia-Lin Hsu, Chun-Ta Huang, Jann-Yuan Wang, Shie-Liang Hsieh, Chong-Jen Yu, Li-Na Lee, Pan-Chyr Yang

**Affiliations:** 1 Department of Traumatology, National Taiwan University Hospital, Taipei, Taiwan; 2 Graduate Institute of Clinical Medicine, College of Medicine, National Taiwan University, Taipei, Taiwan; 3 Department of Internal Medicine, National Taiwan University Hospital, # 7, Chung-Shan South Road, Taipei 100, Taiwan; 4 Institute of Microbiology and Immunology, National Yang-Ming University, Taipei, Taiwan; 5 Institute of Clinical Medicine & Infection and Immunity Center, National Yang-Ming University, Taipei, Taiwan; 6 Department of Laboratory Medicine, National Taiwan University Hospital, Taipei, Taiwan

**Keywords:** Apoptosis, Decoy receptor 3, Latent tuberculosis infection, Lipoxin, Prostaglandin E2, Tuberculosis

## Abstract

**Background:**

Apoptosis-associated biomarkers are rarely studied, especially their role in predicting the development of tuberculosis (TB) from latent TB infection and in prognostication.

**Methods:**

Patients with TB and interferon-gamma release assay (IGRA)-positive and IGRA-negative family contacts were evaluated to analyze changes in apoptosis-associated serum biomarkers, which included decoy receptor 3 (DcR3), prostaglandin 2 (PGE2), and lipoxin. The prognostic implications of these serum biomarkers were also analyzed.

**Results:**

One hundred TB patients and 92 IGRA-negative and 91 IGRA-positive family contacts were recruited. The DcR3 and PGE2 levels decreased from the IGRA-negative group to the IGRA-positive group, and peaked in the TB group. Lipoxin decreased to trough in the TB group. The three apoptosis serum markers and age were independent factors discriminating active TB from latent TB infection. In active TB, older age, co-morbidity, and higher serum DcR3 and monocyte chemotactic protein (MCP)-1 were independently associated with poorer six-month survival.

**Conclusion:**

Apoptosis-associated serum biomarkers change along with the status of *Mycobacterium tuberculosis* infection. In close contacts with positive IGRA, high DcR3 and PGE2 and low lipoxin may increase the probability of active TB. Older age, co-morbidity, and high DcR3 and MCP-1 levels might be important prognostic factors that warrant further investigation.

## Background

Tuberculosis (TB) remains one of the most important infectious diseases worldwide. According to estimates of the World Health Organization (WHO), 3 hundred million people were infected by *Mycobacterium tuberculosis* and 30 million people died of TB from 2001 to 2010 [1,2]. In Taiwan, the incidence of TB remains high (58 per 100,000 population in 2009) [3]. To prevent further transmissions, early diagnosis and timely treatment are the most important strategies [4,5].

Patients infected with *M. tuberculosis* will develop latent TB infection (LTBI), a major source of active TB [6]. Previous studies show that about 10% of LTBI patients will develop the active disease and this risk is even higher in immuno-compromised hosts [7,8]. Nowadays, LTBI is detected by tuberculin skin test and by interferon-gamma release assay (IGRA), which measures T-cell response against tuberculous bacilli-specific antigen [9-11]. In particular, IGRA is reportedly more accurate in diagnosing LTBI and as an indicator of progression to active TB [12]. However, factors predicting the development of active TB from LTBI remain ineffective. Active TB is still identified mainly by traditional chest imaging and mycobacteriologic study.

In the pathogenesis of TB, macrophages are the first-line of defense as the TB bacilli enter the airways [13]. Phagocytosis of the bacilli is then performed by phagocytic antigen-presenting cells in the lungs. However, *M. tuberculosis* has several mechanisms for persisting in human tissues [14-16]. For instance, necrosis of *M. tuberculosis*-infected macrophages as the dominant form of cell death instead of apoptosis [15,17] prevents the removal of intracellular bacilli and the more efficient induction of inflammation [18].

Apoptosis-associated markers, including Decoy receptor-3 (DcR-3), lipoxin, and prostaglandin E2 (PGE2) have rarely been investigated for discriminating active TB from LTBI in clinical practice. Among them, DcR3 is a receptor of the tumor necrosis factor (TNF) receptor super-family, existing as a soluble receptor for Fas ligand, a well-known marker in the extrinsic pathway of apoptosis, and is considered an immuno-modulator [19]. The latter two lipid markers antagonize macrophage apoptosis in an animal model when infected with *M. tuberculosis*[17,20]. Thus, this prospective study enrolled active TB cases and IGRA-positive and IGRA-negative family contacts and compared serum apoptosis-associated markers to identify potentially discriminative diagnostic and prognostic factors.

## Methods

### Enrolment of patients and contacts

This prospective study was conducted in a tertiary-care referral center in northern Taiwan and its branch, a local teaching hospital in southern Taiwan. The Research Ethics Committee of National Taiwan University Hospital approved the study (No. 9561707008). From January 2009 to June 2011, adult patients (age >20 years) with culture- or histology-confirmed active TB were prospectively identified [5]. After providing informed consent, they received peripheral blood sampling before (TB at diagnosis) and one month after anti-TB treatment (TB under treatment) for measurement of serum biomarker levels.

Family contacts with known results of an interferon-gamma release assay (IGRA)-T-SPOT*.TB* (Oxford Immunotech Ltd, Oxford, UK) were also enrolled. Family contacts with negative T-SPOT.*TB* were considered the non-infection group whereas family contacts with positive T-SPOT.*TB* were considered the LTBI group. The contacts received chest radiography and mycobacteriologic study (acid-fast smear and mycobacterial culture) from 3 sputum samples to exclude the possibility of active TB [5,21].

Patients and contacts with human immunodeficiency virus infection and those with bleeding tendency were excluded. Contacts with symptoms compatible with acute infection were also excluded.

### Measurements of biomarkers

Assays for apoptosis-associated markers, including lipid markers (lipoxin and prostaglandin E [PGE]-2) and decoy receptor (DcR)-3, cytokines (interleukin [IL]-6, IL-10, tumor necrosis factor [TNF]-alpha, and interferon [IFN]-gamma), chemokines (monocyte chemotactic protein [MCP]-1, macrophage inflammatory protein [MIP]-1alpha, and MIP-1beta) were performed using serum samples in one batch. All samples were performed in a random order by a technician blinded to the clinical diagnosis. The Bioplex Multiplex Suspension Array System (Bio-Rad *Laboratories Taiwan Ltd.*) [22] was used for all chemokines and cytokines, while the ELISA method was used for lipoxin (Biosource, California, US), PGE2, and DcR3 (R&D Systems Europe, Abingdon, UK).

The lower limits of detection for IL-6, IL-10, IFN-gamma, TNF-alpha, MCP-1, MIP-1alpha, MIP-1beta, DcR3, lipoxin and PGE2 were 1.23, 1.81, 1.8, 4.72, 1.61, 1.4, 1.4, 93.7, 780, and 7.8 pg/ml, respectively. If serum levels of a sample were below the detection range, it was noted as half of the lower limit, i.e. 0.615, 0.905, 0.9, 2.36, 0.805, 0.7, 0.7, 46.85, 390, and 3.9 pg/ml, respectively.

### Data collection

Clinical data, including age, sex, underlying co-morbidities, history of pulmonary TB, laboratory data, and results of acid-fast smear, mycobacterial culture, and drug susceptibility test, as well as anti-TB treatment course and outcomes were all recorded in a standardized case report form. Chest imaging was reviewed and noted as in a previous study [23]. The TB patients were followed-up for at least six months after anti-TB treatment or until death or loss to follow-up.

### Statistical analysis

Serum levels of biomarkers among TB patients and family contacts were compared. Inter-group differences were analyzed by independent sample *t* test or one-way analysis of variance (ANOVA) for numerical variables, and by *chi*-square test for categorical variables. Multivariate logistic regression analysis was used to identify factors associated with TB among TB patients and family contacts with LTBI. For TB patients, survival curves were generated using the Kaplan-Meier method and compared using the log-rank test. Cox proportional hazards regression analysis was used to identify factors associated with six-month survival.

In the stepwise variable selection procedure, all potential predictors were included. A two-sided *p*<0.05 was considered significant. The discriminative power of each biomarker for 1) active TB from LTBI and 2) mortality from survival in TB patients was analyzed using the receiver operating characteristic (ROC) curve and area under the curve (AUC). The optimal cut-off value, defined as the one with the least (1 - sensitivity)^2^ + (1 - specificity)^2^, was used to calculate sensitivity and specificity [24]. All analyses were performed using the SPSS (Version 13.0, Chicago, IL).

## Results

During the study period, 100 TB patients (TB at diagnosis group), 92 IGRA-negative (non-infection group), and 91 IGRA-positive (LTBI group) family contacts were enrolled. The TB patients were significantly older, with male predominance, and had higher proportion of prior TB history and underlying co-morbidity compared to the family contacts (Table 1). Among the 100 TB patients, 55 received follow-up blood sampling one month after anti-TB treatment (TB under treatment group).

**Table 1:** Clinical characteristics of patients with tuberculosis (TB) and family contacts by responses to interferon-gamma release assay

	**IGRA-negative contacts n=92**	**IGRA-positive contacts n=91**	**TB patients n=100**	***p *value**
Age (years)	44.6 [16.9]	51.3 [15.6]	70.3 [16.2]	<0.001
Male sex	32 (35%)	33 (36%)	62 (62%)	<0.001
History of TB	0	0	10 (10%)	<0.001
Co-morbidity	3 (3%)	7 (8%)	39 (39%)	<0.001
Diabetes mellitus	2 (2%)	5 (6%)	30 (30%)	<0.001
Malignancy	1 (1%)	3 (3%)	15 (15%)	<0.001
Liver cirrhosis	0	0	2 (2%)	0.158

Data are no. (%) or mean [standard deviation].

Between the non-infection and LTBI groups, there was no significant difference in serum levels of biomarkers except for MCP-1 (27.86 vs. 18.30 pg/ml, *p*=0.039). Between the LTBI and TB at diagnosis groups, IL-6 (2.7 vs. 64 pg/ml, *p*=0.018), MCP-1 (18.30 vs. 34.05, *p*=0.043), DcR3 (0.64 vs. 3.97 ng/ml, *p*<0.001), and PGE2 (0.31 vs. 3.04, *p*=0.001) were significantly higher in the latter, whereas lipoxin (3.391 vs. 1.73, *p*=0.008) was significantly higher in the former. All cytokines, DcR3, lipoxin, and PGE2 levels were not significantly different between TB patients with malignancy and those without. Serum levels of these markers were also not significantly different between the TB at diagnosis and TB under treatment groups (see online Additional file 1).

**Figure 1:**
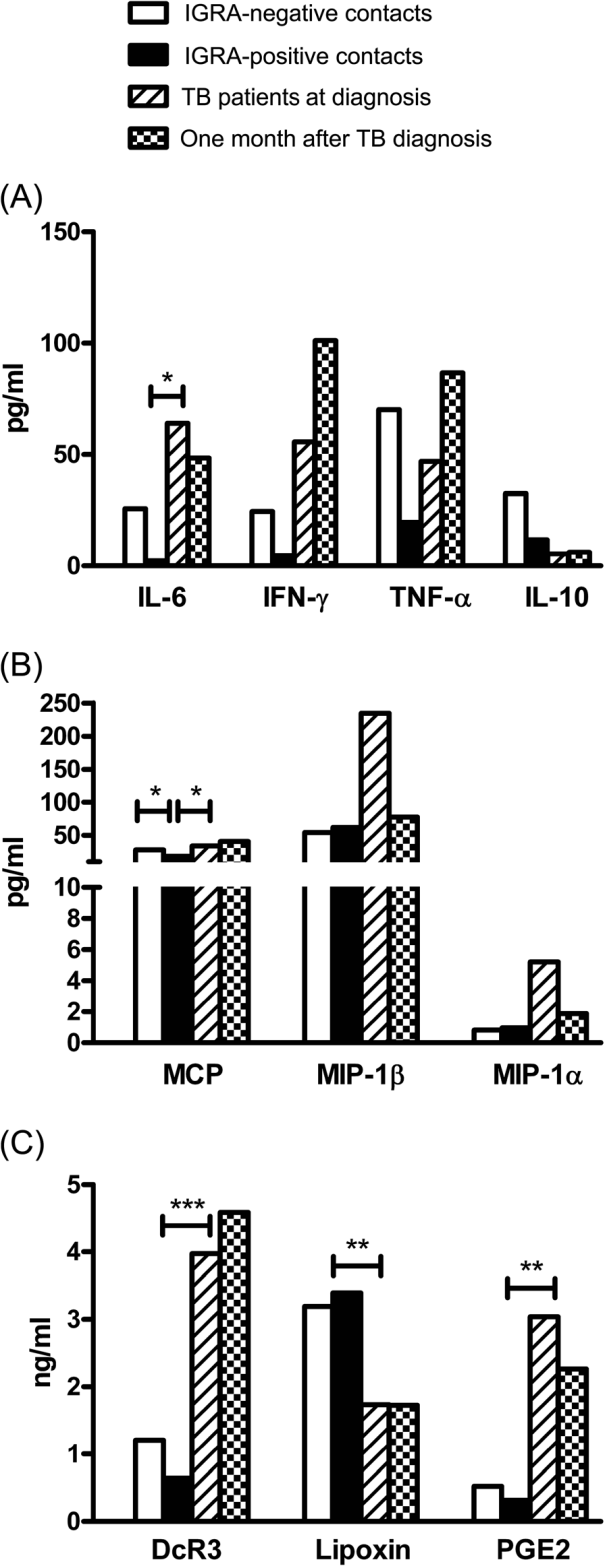
**Histogram shows serum levels of (A) cytokines, (B) chemokines, and (C) apoptosis-associated markers among family contacts and patient with tuberculosis. **Dx, diagnosis; Tx, treatment; *p<0.050; **p<0.010; ***p<0.001.

Among the four groups (i.e., non-infection group, LTBI group, TB at diagnosis group, and TB under treatment group), inflammatory cytokines, including IL-6, TNF-alpha, and IFN-gamma decreased from the non-infection group to the LTBI group, and then increased in the TB patients (Figure 1A). In contrast, IL-10 showed a continuous decreasing trend (statistically not significant). Regarding chemokines, the trend of MCP-1 was comparable to those of inflammatory cytokines but the trends of MIP-1alpha and MIP-1beta increased gradually from the non-infection group to the LTBI group to the TB group (Figure 1B). As for apoptosis-associated markers, the trends of DcR3 and PGE2 were similar to those of inflammatory cytokines but lipoxin had an opposite trend (Figure 1C).

**Figure 2:**
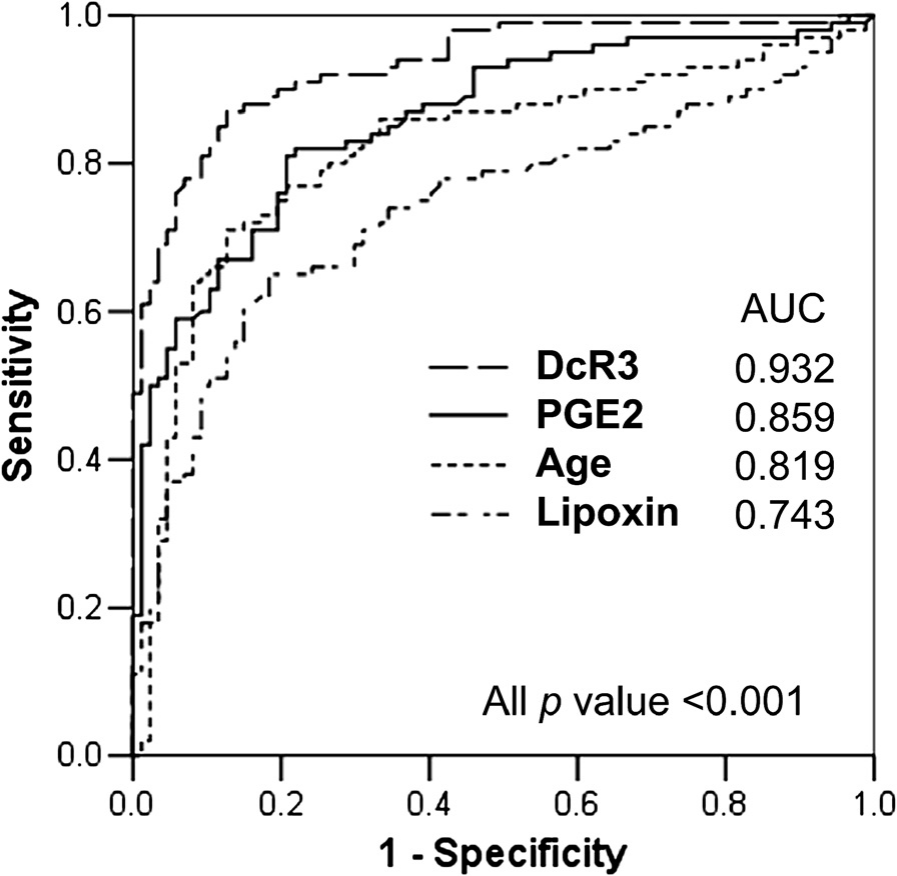
Receiver operating characteristic curves of factors independently associated with tuberculosis among interferon-gamma release assay (IGRA)-positive contacts and patients with tuberculosis.

Multivariate logistic regression analysis for the LTBI and TB at diagnosis groups revealed that age and serum DcR3, PGE2, and lipoxin levels were independently associated with active TB (Table 2). The results were similar in the sub-population analysis focusing on subjects without malignancy (*p*=0.007, *p*<0.001, *p*=0.004, *p*=0.017, respectively). The AUC under the ROC curves of age, DcR3, PGE2, and lipoxin was 0.819, 0.932, 0.859, and 0.743, respectively (Figure 2). The optimal cut-off values were 67 years old (sensitivity 71%; specificity 87%), 1.14 ng/ml (sensitivity 87%; specificity 87%), 0.35 ng/ml (sensitivity 81%; specificity 79%), and 1.82 ng/ml (sensitivity 65%; specificity 82%), respectively. A combination of three biomarkers was used to predict the probability of active TB disease (Table 3) and the presence of any two provided a sensitivity of 99% and specificity of 72%.

**Table 2:** Factors significantly associated with tuberculosis (TB) in multivariate logistic regression analysis among contacts with latent TB infection and TB patients

**Characteristics**	***p *value**	**OR (95% C.I.)**
Age, per 1 year increment	0.003	1.07 (1.02-1.11)
DcR 3, per 1 ng/mL increment	<0.001	11.91 (4.03-35.17)
Lipoxin, per 1 ng/mL increment	<0.001	0.39 (0.23-0.66)
PGE2, per 1 ng/mL increment	<0.001	21.71 (4.66-101.28)

Abbreviations: TB, tuberculosis; DcR 3, decoy receptor 3; PGE2, prostaglandin E2.

**Table 3:** Predicting the presence of tuberculosis (TB) by biomarkers

**Conditions**	**No. of matched subjects**	**TB patients**	**Sensitivity**	**Specificity**	**Positive predictive value**	**Negative predictive value**
All	59	51	51%	92%	86%	65%
Any two	127	99	99%	72%	78%	99%
Anyone	140	99	99%	59%	71%	98%

The three clinical factors included were Decoy receptor 3 (DcR3) >1.14 ng/mL, prostaglandin E2 (PGE2) >0.35 ng/mL, and lipoxin <1.82 ng /mL.

Among the 100 TB patients, 21 (21%) died (of any cause) during the study. Comparing the clinical characteristics, survivors were younger and less likely to have underlying co-morbidity. Non-survivors had higher serum MCP-1 (*p*=0.012) and DcR3 (*p*=0.078) levels (Table 4).

**Table 4:** Clinical characteristics, laboratory results, radiologic findings and disease extent among patients with tuberculosis (TB)

	**Survivors n=79**	**Non-survivors n=21**	***p *value**
Age (years)	68.2 [16.9]	78.3 [9.6]	0.010
Male sex	48 (61%)	14 (67%)	0.620
History of TB	6 (8%)	4 (19%)	0.120
Co-morbidity	27 (34%)	12 (57%)	0.055
Diabetes mellitus	24 (30%)	6 (29%)	0.872
Malignancy	6 (8%)	9 (43%)	<0.001
Liver cirrhosis	0	2 (10%)	0.006
Diagnosis evidence			0.051
Culture positive	79 (100%)	20 (95%)	
Pathology positive	0	1 (5%)	
Mtb resistance			
INH resistance	8 (10%)	0	0.128
RIF resistance	0	1 (5%)	0.051
Sputum AFS-positive	52 (66%)	11 (52%)	0.257
Radiographic pattern			
Upper lung lesion	53 (67%)	13 (62%)	0.656
Cavitation	11 (14%)	1 (5%)	0.251
Disease extent			0.653
Pulmonary	64 (81%)	18 (86%)	
Extra-pulmonary only	3 (4%)	0	
Mixed	12 (15%)	3 (14%)	
Blood tests			
Leukocyte, (/μL)	9609 [4880]	10297 [4207]	0.542
Interleukin-6	54.9 [257.3]	98.2 [196.6]	0.408
Interleukin-10	5.6 [23.1]	4.7 [12.2]	0.814
Interferon-gamma	66.3 [472.7]	15.4 [38.9]	0.347
TNF-alpha	50.8 [375.5]	32.7 [123.7]	0.720
MCP-1	25.0 [50.1]	68.2 [116.1]	0.012
MIP-1alpha	6.1 [29.8]	1.8 [2.6]	0.210
MIP-1beta	245.4 [1274.7]	193.3 [366.2]	0.752
Decoy receptor 3	3.6 [3.8]	5.3 [3.7]	0.078
Lipoxin	1.7 [1.5]	1.7 [1.2]	0.939
Prostaglandin E2	2.9 [8.1]	3.4 [7.0]	0.793

Data are no. (%) or mean [standard deviation].

Abbreviations: MCP, monocyte chemotactic protein; MIP, macrophage inflammatory protein; TNF, tumor necrosis factor.

**Figure 3:**
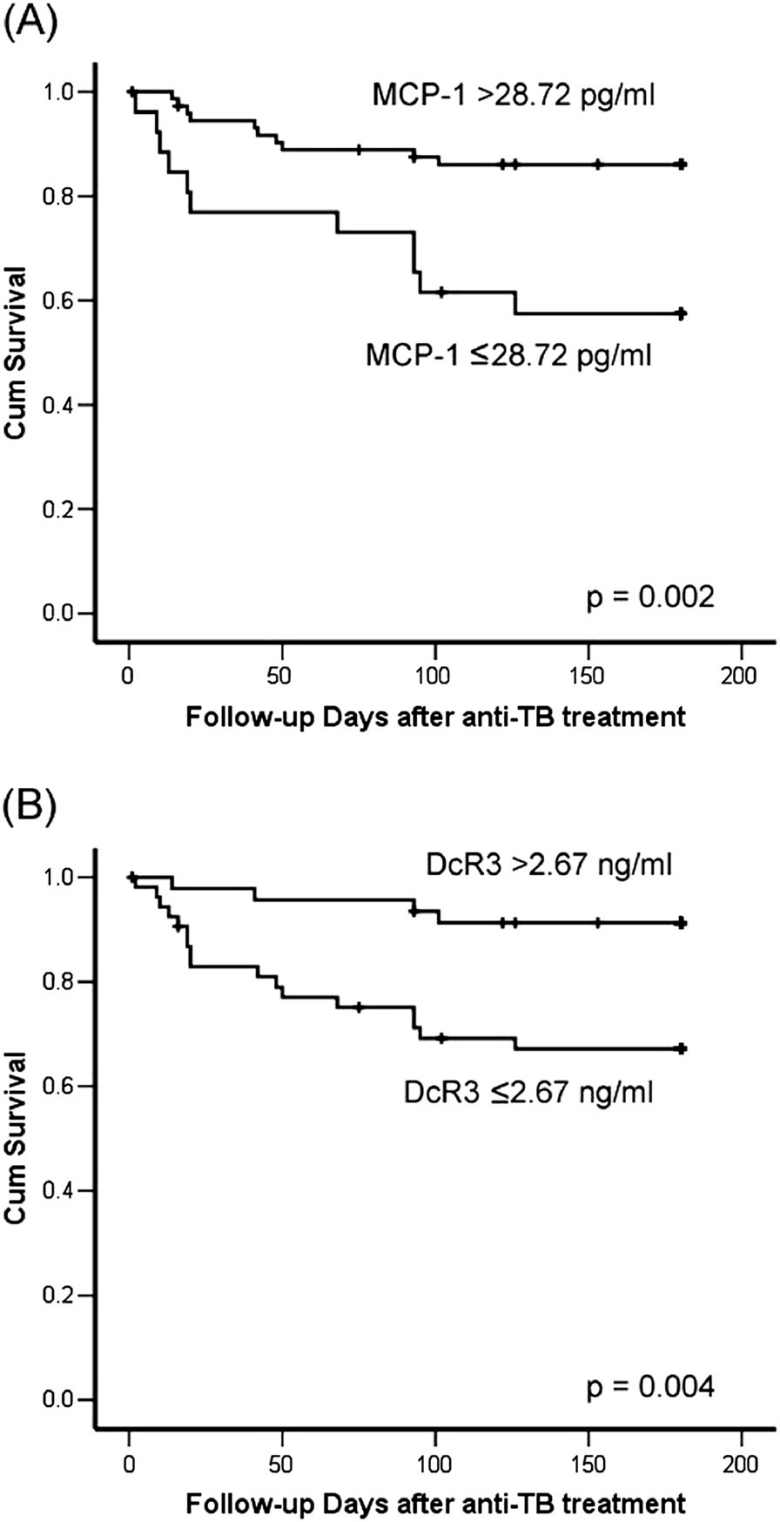
Survival curves plotted using the Kaplan-Meier method for patients with tuberculosis and different serum levels of (A) MCP-1 and (B) DcR3.

Using the optimal cut-off values determined by ROC curve analysis, the Kaplan-Meier survival curves of TB patients according to serum MCP-1 (>28.72 *vs*. ≤28.72 pg/ml) and DcR3 (>2.67 *vs*. ≤2.67 ng/ml) levels were plotted (Figure 3). The *p* values compared by log-rank test were 0.002 and 0.004, respectively. Multivariate Cox regression analysis revealed that age, co-morbidity, and serum MCP-1 and DcR3 levels were independent factors predicting six-month survival (Table 5).

**Table 5:** Factors independently associated with worse six-month survival in patients with tuberculosis, by Cox proportional hazards regression analysis

**Characteristics**	***p *value**	**HR (95% C.I.)**
Age, per 1 year increment	0.008	1.072 (1.018-1.129)
Co-morbidity	0.007	3.856 (1.450-10.256)
DcR 3, per 1 ng/mL increment	0.046	1.089 (1.001-1.183)
MCP, per 1 pg/mL increment	<0.001	1.008 (1.004-1.013)
PGE2, per 1 ng/mL increment	0.063	1.059 (0.997-1.125)

Abbreviations: DcR 3, Decoy receptor 3; MCP, monocyte chemotactic protein; PGE2, Prostaglandin E2.

## Discussion

Using four groups (i.e., non-infection group, LTBI group, TB at diagnosis group, and TB under treatment group) of data that represent the different stages in the development of active TB disease, the present study reveals that apoptosis-associated biomarkers change significantly. Levels of DcR3 and PGE2 are elevated but lipoxin is decreased when patients have active TB compared to latent infection. Combinations of the three biomarkers and age may be useful and promising tools in clinical practice to predict the development of active TB among family contacts with LTBI. As for TB patients, age and underlying co-morbidity, and serum DcR3 and MCP-1 levels at the time of diagnosis are significantly associated with six-month survival.

During latent infection, *M. tuberculosis* has several mechanisms to protect itself from human immunity [14-16]. One is the suppression of apoptosis of infected macrophages because this will result in the removal of intracellular bacilli and induction of inflammation [18,25]. *M. tuberculosis* also promotes its replication by inhibiting the apoptosis of infected macrophages [18]. Macrophage death toward necrosis instead of apoptosis is therefore a considerably advantageous evasion mechanism of *M. tuberculosis*[26].

Later, there are overwhelming systemic inflammatory responses when active disease develops. Thus, levels of inflammatory cytokines decrease when subjects progress from non-infection status to latent infection, and peak in the active TB disease status. Although DcR3 and PGE2 levels have a similar trend, DcR3 may be a downstream (passive) responder to inflammation-related apoptosis and enhance the signal pathway. PGE2 plays a relatively active role in the upstream of apoptosis [19,27,28]. In addition, the trend of lipoxin shows its function of antagonizing PGE2 in apoptosis control.

After *M. tuberculosis* infection, macrophages are activated locally and chemokines increase, leading to a gradual increase in serum MIP level from non-infection and LTBI to active TB. However, MCP-1 recruits monocytes and T lymphocytes later during the granulomatous response [29] and may not significantly increase during LTBI. This explains the similar trend of change as inflammatory cytokines.

Apoptosis-associated biomarkers, rather than inflammatory cytokines, are independent factors in predicting active TB. They are of practical importance yet remain largely unexplored. This is probably because inflammatory cytokines may be influenced by multiple factors during inflammation, whereas apoptosis-associated biomarkers are much simpler in their pathways and have less influence. Although apoptosis-associated markers may be confounded by underlying co-morbidity, the association between these markers and active TB is probably independent of underlying co-morbidity, based on the results of the current analyses.

Among the apoptosis-associated biomarkers, DcR3 seems to be the most associated with immune cells [19,28,30]. It has the potential to discriminate between latent and active TB using the ROC curve analysis. From a public health consideration, a test with high sensitivity for predicting the development of active TB from LTBI is most useful in preventing delayed TB diagnosis and further transmission. If 99% of active TB cases can be identified by DcR3 >1.14 ng/ml plus PGE2 >0.35 ng/ml, these will be useful as screening criteria.

The six-month survival of TB patients may be confounded by numerous factors and is difficult to predict simply based on characteristics at diagnosis. Many influential factors may be missed in the analysis. Nonetheless, the results imply that baseline health conditions and extent of immune response to TB have prognostic importance. DcR3 has been reported as an immuno-modulator that skews immune response into type II macrophages but also induces dendritic cell apoptosis [28,30]. Hence, it may be a promising indicator of overwhelming inflammatory response for destruction during active TB. As a potent recruiting signal for monocytes and T cells [31], higher serum MCP-1 level may imply more severe granulomatous inflammation [29]. This is the first time that MCP-1 level in TB patients is demonstrated as a promising tool for outcome prediction.

This study has some limitations. First, inter-group comparison is performed in different individuals and may be biased by individual variations. Disease processes other than TB, like cancer, may influence serum levels of biomarkers. Second, biomarker responses after *in vitro* stimulation are not assayed. Thus, it cannot be simply concluded that the measured levels of biomarkers in study subjects are solely due to immune response against *M. tuberculosis*. Third, the study was conducted in a tertiary referral center and a regional teaching hospital. The high prevalence of underlying co-morbidities in TB patients may change the serum levels of biomarkers and lead to a relatively high mortality rate. This limits the external generalization of the study results. Furthermore, due to the small number of follow-up samples, firm conclusions cannot be made for the biomarker levels after treatment.

## Conclusion

In conclusion, apoptosis-associated markers significantly correlate with the status of *M. tuberculosis* infection. High DcR3 level, high PGE2 level, and low lipoxin level may be highly sensitive criteria for identifying active TB cases among contacts with LTBI. Prognosis is poorer in TB patients with older age, underlying co-morbidity, and high serum DcR3 and MCP-1 levels.

## Competing interests

All of the authors declare no financial, professional, or other personal interests of any nature or kind in any related product, service, and/or company.

## Authors’ contribution

LLN and WJY conceived the study. WJY, SCC, HCL and HCT were involved in the sample and clinical data collection. SCC, WJY, HCL, LLN and YPC participated in data analysis and in manuscript writing, together with WMF and HSL. YCJ and WJY were directors responsible for the study organization. All authors read and approved the final manuscript.

## Pre-publication history

The pre-publication history for this paper can be accessed here:

http://www.biomedcentral.com/1471-2334/13/45/prepub

## Supplementary Material

Additional file 1 The details of serum markers in patients with tuberculosis and the family contacts. Click here for file
