# Supplementary material for: Apoptosis-associated biomarkers in tuberculosis: promising for diagnosis and prognosis prediction
Source: BMC Infect Dis. 2013 Jan 28;13:45. doi: 10.1186/1471-2334-13-45 (PMC3566962; doi:10.1186/1471-2334-13-45)
Supplement: Additional file 1 — The details of serum markers in patients with tuberculosis and the family contacts. [file 1471-2334-13-45-S1.doc]

**Table E1.** Serum markers in patients with tuberculosis and the family contacts

|  | IGRA-negative  n=92 | *p* value* | IGRA-positive  n=91 | *p* value^#^ | Tuberculosis (TB) | | |
| --- | --- | --- | --- | --- | --- | --- | --- |
|  |  |  |  |  | At diagnosis  n=100 | *p* value^ⱡ^ | After 1-month Tx  n=55 |
| Inerleukin-6, pg/ml | 25.8 [205.8] | 0.285 | 2.7 [12.8] | 0.018 | 64.0 [245.5] | 0.620 | 48.4 [145.6] |
| Interferon-gamma, pg/ml | 24.54 [192.0] | 0.325 | 4.70 [13.2] | 0.249 | 55.65 [420.5] | 0.615 | 101.16 [591.6] |
| TNF-alpha, pg/ml | 70.20 [567.3] | 0.404 | 19.69 [108.3] | 0.445 | 46.99 [338.0] | 0.582 | 86.73 [471.1] |
| Interleukin-10, pg/ml | 32.6 [275.7] | 0.490 | 11.9 [79.2] | 0.453 | 5.4 [21.2] | 0.857 | 6.2 [26.0] |
| MCP-1, pg/ml | 27.86 [38.5] | 0.039 | 18.30 [21.2] | 0.043 | 34.05 [70.8] | 0.684 | 40.70 [108.7] |
| MIP-1alpha, pg/ml | 0.83 [0.5] | 0.155 | 0.97 [0.8] | 0.129 | 5.21 [26.5] | 0.126 | 1.88 [3.8] |
| MIP-1beta, pg/ml | 54.31 [36.8] | 0.274 | 61.68 [52.8] | 0.152 | 234.41 [1143.5] | 0.175 | 77.69 [67.6] |
| Decoy receptor 3, ng/ml | 1.21 [3.0] | 0.082 | 0.64 [0.6] | <0.001 | 3.97 [4.0] | 0.286 | 4.59 [3.2] |
| Prostaglandin E2, ng/ml | 0.52 [2.3] | 0.388 | 0.31 [0.4] | 0.001 | 3.04 [7.8] | 0.360 | 2.27 [3.3] |
| Lipoxin, ng/ml | 3.19 [4.8] | 0.801 | 3.39 [6.0] | 0.008 | 1.73 [1.4] | 0.968 | 1.73 [1.2] |

Data, except for the *p* values, are the mean [standard deviation]

Abbreviations: IGRA, interferon-gamma release assay; MCP, monocyte chemotactic protein; MIP, macrophage inflammatory protein; TNF, tumor necrosis factor; Tx, anti-tuberculosis treatment

**p* value of comparison between IGRA-positive and IGRA-negative contacts

^#^*p* value of comparison between IGRA-positive contacts and TB patients

^ⱡ^*p* value of comparison between data at TB diagnosis and data after one-month treatment
